# *trans*-Dibromidobis(tri-*p*-tolyl­arsine)palladium(II)

**DOI:** 10.1107/S1600536809043372

**Published:** 2009-10-28

**Authors:** Leo Kirsten, Gideon Steyl, Andreas Roodt

**Affiliations:** a Department of Chemistry, University of the Free State, PO Box 339, Bloemfontein 9300, South Africa

## Abstract

In the title compound, [PdBr_2_(C_21_H_21_As)_2_], the Pd^II^ ion, residing on a centre of symmetry, is coordinated by two As donor atoms [Pd—As = 2.4276 (2) Å] and two Br anions [Pd—Br = 2.4194 (2) Å] in a distorted square-planar geometry [Br—Pd—As = 87.786 (7)°]. A weak intra­molecular C—H⋯Br inter­action occurs. In the crystal structure, inter­molecular C—H⋯Br inter­actions are observed.

## Related literature

For similar palladium complexes containing arsine and bromido derivatives, see: Kirsten & Steyl (2009 ▶) and references therein.
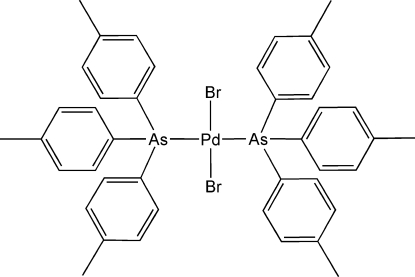


## Experimental

### 

#### Crystal data

[PdBr_2_(C_21_H_21_As)_2_]
*M* *_r_* = 962.82
Monoclinic, 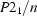

*a* = 10.2435 (4) Å
*b* = 18.2139 (8) Å
*c* = 10.7509 (4) Å
β = 106.185 (2)°
*V* = 1926.34 (13) Å^3^
*Z* = 2
Mo *K*α radiation
μ = 4.29 mm^−1^
*T* = 100 K
0.35 × 0.29 × 0.26 mm

#### Data collection

Bruker X8 APEXII 4K Kappa CCD diffractometer
Absorption correction: multi-scan (*SADABS*; Bruker, 1998 ▶) *T*_min_ = 0.258, *T*_max_ = 0.330
25665 measured reflections
4194 independent reflections
3862 reflections with *I* > 2σ(*I*)
*R*_int_ = 0.037

#### Refinement

*R*[*F* ^2^ > 2σ(*F* ^2^)] = 0.019
*wR*(*F* ^2^) = 0.046
*S* = 1.04
4194 reflections
217 parameters
H-atom parameters constrained
Δρ_max_ = 0.51 e Å^−3^
Δρ_min_ = −0.44 e Å^−3^

### 

Data collection: *APEX2* (Bruker, 2005 ▶); cell refinement: *SAINT-Plus* (Bruker, 2004 ▶); data reduction: *SAINT-Plus* and *XPREP* (Bruker 2004 ▶); program(s) used to solve structure: *SHELXS97* (Sheldrick, 2008 ▶); program(s) used to refine structure: *SHELXL97* (Sheldrick, 2008 ▶); molecular graphics: *DIAMOND* (Brandenburg & Putz, 2006 ▶); software used to prepare material for publication: *SHELXL97*.

## Supplementary Material

Crystal structure: contains datablocks I, global. DOI: 10.1107/S1600536809043372/cv2631sup1.cif

Structure factors: contains datablocks I. DOI: 10.1107/S1600536809043372/cv2631Isup2.hkl

Additional supplementary materials:  crystallographic information; 3D view; checkCIF report

## Figures and Tables

**Table 1:** Hydrogen-bond geometry (Å, °)

*D*—H⋯*A*	*D*—H	H⋯*A*	*D*⋯*A*	*D*—H⋯*A*
C32—H32⋯Br	0.95	2.95	3.764 (2)	144
C35—H35⋯Br^i^	0.95	2.94	3.787 (2)	149

Symmetry code: (i) 
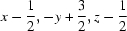
.

**Fig. 1.**
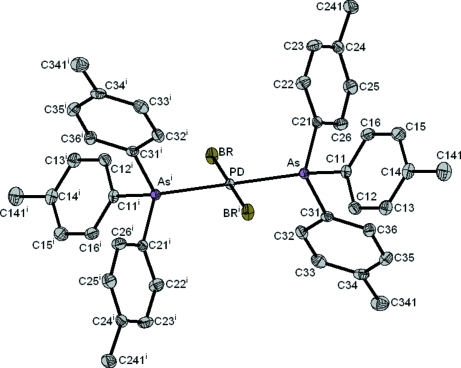
The molecular structure of (I) showing the atomic numbering and 30% probability displacement ellipsoids [symmetry code: (i) -x,-y,-z].. H atoms have been omitted for clarity.

## supplementary crystallographic information

### Comment

In continuation of our study of square-planar palladium complexes containing arsine donor and bromido ligands (Kirsten & Steyl, 2009) we present here the title compound, (I).

The molecule of (I) (Fig. 1), is centrosymmetric, so pairs of equivalent arsino donor ligands lie *trans* to one another in a slightly distorted square-planar geometry, with the *cis* angles deviating from 90° by less than 3° [Br—Pd—As 87.786 (7)°]. A staggered conformation of the two triphenyl arsine moieties is observed, supported by the Br—Pd—As—C_n_ torsion angles of 160.99 (6)° (C_n_=C11), 37.93 (6)° (C_n_=C12) and -78.75 (6)° (C_n_=C13), respectively.

Weak intra- and intermolecular hydrogen-bonding interactions are observed between the Br and the hydrogen atoms of the triphenylarsine ligands (Table 1). The effect of the methyl substituent on the *para* position of the phenyl rings has no significant effect on the crystallization mode of the complex when compared the the closely related triphenylarsine complex (Kirsten & Steyl, 2009). The rms error of 0.173 Å indicates the iso-structurality of the two complexes (the title complex superimposed with the triphenylarsine complex (Kirsten & Steyl, 2009) including all the atoms except the methyl substituents and the hydrogen atoms).

### Experimental

The title compound was synthesized by the addition of As(pTol)_3_ (20 mg, 0.0059 mmol) to an acetone solution (15 cm^3^) of Pd(Br)_2_(COD) (10 mg, 0.027 mmol). Crystals suitable for diffraction were obtained by slow evaporation of the reaction mixture (yield 18 mg, 71%).

### Refinement

All H atoms were positioned geometrically (C—H = 0.95–0.98 Å) and allowed to ride on their parent atoms, with *U*_iso_(H) = 1.2–1.5 *U*_eq_ of the parent atom.

### Crystal data

**Table:**

[PdBr_2_(C_21_H_21_As)_2_]	*F*(000) = 952
*M**_r_* = 962.82	*D*_x_ = 1.660 Mg m^−^^3^
Monoclinic, *P*2_1_/*n*	Mo *K*α radiation, λ = 0.71073 Å
Hall symbol: -P 2yn	Cell parameters from 6121 reflections
*a* = 10.2435 (4) Å	θ = 2.2–28.3°
*b* = 18.2139 (8) Å	µ = 4.29 mm^−^^1^
*c* = 10.7509 (4) Å	*T* = 100 K
β = 106.185 (2)°	Cuboid, orange
*V* = 1926.34 (13) Å^3^	0.35 × 0.29 × 0.26 mm
*Z* = 2	

### Data collection

**Table:**

Bruker X8 APEXII 4K Kappa CCD diffractometer	4194 independent reflections
Radiation source: fine-focus sealed tube	3862 reflections with *I* > 2σ(*I*)
graphite	*R*_int_ = 0.037
Detector resolution: 512 pixels mm^-1^	θ_max_ = 27.0°, θ_min_ = 2.2°
φ and ω scans	*h* = −13→13
Absorption correction: multi-scan (*SADABS*; Bruker, 1998)	*k* = −23→23
*T*_min_ = 0.258, *T*_max_ = 0.330	*l* = −13→13
25665 measured reflections	

### Refinement

**Table:**

Refinement on *F*^2^	Primary atom site location: structure-invariant direct methods
Least-squares matrix: full	Secondary atom site location: difference Fourier map
*R*[*F*^2^ > 2σ(*F*^2^)] = 0.019	Hydrogen site location: riding model
*wR*(*F*^2^) = 0.046	H-atom parameters constrained
*S* = 1.03	*w* = 1/[σ^2^(*F*_o_^2^) + (0.0166*P*)^2^ + 1.4379*P*] where *P* = (*F*_o_^2^ + 2*F*_c_^2^)/3
4194 reflections	(Δ/σ)_max_ = 0.001
217 parameters	Δρ_max_ = 0.51 e Å^−^^3^
0 restraints	Δρ_min_ = −0.44 e Å^−^^3^

### Special details

**Table:**

Geometry. All e.s.d.'s (except the e.s.d. in the dihedral angle between two l.s. planes) are estimated using the full covariance matrix. The cell e.s.d.'s are taken into account individually in the estimation of e.s.d.'s in distances, angles and torsion angles; correlations between e.s.d.'s in cell parameters are only used when they are defined by crystal symmetry. An approximate (isotropic) treatment of cell e.s.d.'s is used for estimating e.s.d.'s involving l.s. planes.
Refinement. Refinement of *F*^2^ against ALL reflections. The weighted *R*-factor *wR* and goodness of fit *S* are based on *F*^2^, conventional *R*-factors *R* are based on *F*, with *F* set to zero for negative *F*^2^. The threshold expression of *F*^2^ > σ(*F*^2^) is used only for calculating *R*-factors(gt) *etc*. and is not relevant to the choice of reflections for refinement. *R*-factors based on *F*^2^ are statistically about twice as large as those based on *F*, and *R*- factors based on ALL data will be even larger.

### Fractional atomic coordinates and isotropic or equivalent isotropic displacement parameters (Å^2^)

**Table:**

	*x*	*y*	*z*	*U*_iso_*/*U*_eq_	
Pd	0.5000	0.5000	0.5000	0.01048 (5)	
Br	0.691876 (19)	0.534223 (11)	0.679231 (18)	0.01856 (6)	
As	0.558809 (18)	0.601507 (10)	0.379536 (18)	0.01109 (5)	
C11	0.48804 (19)	0.60151 (10)	0.19217 (18)	0.0137 (4)	
C12	0.3492 (2)	0.61094 (11)	0.13618 (19)	0.0169 (4)	
H12	0.2897	0.6156	0.1895	0.020*	
C13	0.2977 (2)	0.61356 (11)	0.00240 (19)	0.0182 (4)	
H13	0.2028	0.6201	−0.0351	0.022*	
C14	0.3827 (2)	0.60683 (10)	−0.07756 (19)	0.0180 (4)	
C15	0.5207 (2)	0.59546 (12)	−0.0208 (2)	0.0217 (4)	
H15	0.5799	0.5890	−0.0741	0.026*	
C16	0.5730 (2)	0.59340 (11)	0.1131 (2)	0.0192 (4)	
H16	0.6677	0.5864	0.1507	0.023*	
C141	0.3282 (2)	0.61492 (13)	−0.2220 (2)	0.0263 (5)	
H14A	0.2321	0.6008	−0.2489	0.039*	
H14B	0.3796	0.5831	−0.2648	0.039*	
H14C	0.3372	0.6661	−0.2463	0.039*	
C21	0.75110 (18)	0.61873 (10)	0.40559 (17)	0.0130 (4)	
C22	0.8349 (2)	0.56148 (11)	0.3902 (2)	0.0191 (4)	
H22	0.7985	0.5135	0.3716	0.023*	
C23	0.9715 (2)	0.57403 (12)	0.4019 (2)	0.0204 (4)	
H23	1.0270	0.5348	0.3883	0.025*	
C24	1.02824 (18)	0.64337 (11)	0.43333 (18)	0.0170 (4)	
C25	0.9446 (2)	0.69946 (11)	0.4526 (2)	0.0209 (4)	
H25	0.9821	0.7468	0.4764	0.025*	
C26	0.8071 (2)	0.68784 (11)	0.4377 (2)	0.0184 (4)	
H26	0.7512	0.7274	0.4496	0.022*	
C241	1.1756 (2)	0.65736 (13)	0.4429 (2)	0.0242 (5)	
H24A	1.2274	0.6118	0.4667	0.036*	
H24B	1.2115	0.6948	0.5092	0.036*	
H24C	1.1839	0.6747	0.3591	0.036*	
C31	0.50031 (18)	0.69617 (10)	0.42613 (18)	0.0130 (4)	
C32	0.51153 (19)	0.71093 (11)	0.55552 (19)	0.0172 (4)	
H32	0.5397	0.6734	0.6187	0.021*	
C33	0.4816 (2)	0.78054 (11)	0.5922 (2)	0.0202 (4)	
H33	0.4892	0.7901	0.6808	0.024*	
C34	0.44053 (19)	0.83669 (11)	0.5019 (2)	0.0186 (4)	
C35	0.4280 (2)	0.82081 (11)	0.3726 (2)	0.0201 (4)	
H35	0.3986	0.8581	0.3091	0.024*	
C36	0.45788 (19)	0.75149 (11)	0.33475 (19)	0.0168 (4)	
H36	0.4493	0.7418	0.2460	0.020*	
C341	0.4165 (2)	0.91285 (12)	0.5440 (2)	0.0267 (5)	
H34A	0.3483	0.9374	0.4741	0.040*	
H34B	0.5017	0.9407	0.5640	0.040*	
H34C	0.3838	0.9102	0.6213	0.040*	

### Atomic displacement parameters (Å^2^)

**Table:**

	*U*^11^	*U*^22^	*U*^33^	*U*^12^	*U*^13^	*U*^23^
Pd	0.00953 (10)	0.01177 (10)	0.00909 (10)	−0.00197 (7)	0.00087 (7)	0.00047 (7)
Br	0.01676 (10)	0.02136 (11)	0.01337 (10)	−0.00722 (7)	−0.00274 (7)	0.00204 (7)
As	0.00983 (9)	0.01240 (9)	0.01058 (10)	−0.00086 (7)	0.00209 (7)	0.00121 (7)
C11	0.0163 (9)	0.0128 (9)	0.0115 (9)	−0.0005 (7)	0.0029 (7)	0.0016 (7)
C12	0.0168 (10)	0.0186 (10)	0.0159 (10)	−0.0005 (7)	0.0056 (8)	0.0011 (8)
C13	0.0161 (10)	0.0182 (10)	0.0174 (10)	−0.0022 (7)	0.0000 (8)	0.0012 (8)
C14	0.0259 (11)	0.0130 (9)	0.0129 (10)	−0.0014 (8)	0.0020 (8)	−0.0013 (7)
C15	0.0261 (11)	0.0246 (11)	0.0166 (10)	0.0047 (8)	0.0095 (8)	0.0003 (8)
C16	0.0165 (10)	0.0216 (10)	0.0193 (10)	0.0046 (8)	0.0047 (8)	0.0015 (8)
C141	0.0342 (12)	0.0291 (12)	0.0133 (10)	−0.0012 (9)	0.0029 (9)	−0.0010 (9)
C21	0.0109 (9)	0.0165 (9)	0.0107 (9)	0.0000 (7)	0.0018 (7)	0.0033 (7)
C22	0.0165 (10)	0.0158 (10)	0.0235 (11)	−0.0002 (7)	0.0032 (8)	−0.0012 (8)
C23	0.0149 (10)	0.0226 (11)	0.0237 (11)	0.0053 (8)	0.0051 (8)	0.0007 (9)
C24	0.0115 (9)	0.0263 (11)	0.0124 (9)	0.0000 (8)	0.0022 (7)	0.0052 (8)
C25	0.0184 (10)	0.0177 (10)	0.0264 (11)	−0.0060 (8)	0.0062 (8)	−0.0007 (9)
C26	0.0153 (9)	0.0156 (10)	0.0251 (11)	0.0009 (7)	0.0069 (8)	−0.0010 (8)
C241	0.0128 (10)	0.0345 (12)	0.0245 (11)	−0.0010 (8)	0.0037 (8)	0.0066 (9)
C31	0.0087 (8)	0.0140 (9)	0.0162 (9)	−0.0007 (7)	0.0030 (7)	−0.0008 (7)
C32	0.0170 (9)	0.0207 (10)	0.0146 (10)	−0.0018 (7)	0.0058 (7)	0.0014 (8)
C33	0.0198 (10)	0.0243 (11)	0.0175 (10)	−0.0034 (8)	0.0072 (8)	−0.0045 (8)
C34	0.0115 (9)	0.0195 (10)	0.0254 (11)	−0.0006 (7)	0.0063 (8)	−0.0029 (8)
C35	0.0195 (10)	0.0192 (10)	0.0207 (10)	0.0040 (8)	0.0042 (8)	0.0030 (8)
C36	0.0175 (10)	0.0191 (10)	0.0130 (9)	0.0010 (7)	0.0029 (7)	0.0005 (8)
C341	0.0272 (12)	0.0227 (11)	0.0317 (13)	0.0010 (9)	0.0107 (10)	−0.0056 (9)

### Geometric parameters (Å, °)

**Table:**

Pd—Br^i^	2.4194 (2)	C23—C24	1.392 (3)
Pd—Br	2.4194 (2)	C23—H23	0.9500
Pd—As^i^	2.42761 (19)	C24—C25	1.385 (3)
Pd—As	2.42761 (19)	C24—C241	1.506 (3)
As—C21	1.9363 (18)	C25—C26	1.389 (3)
As—C31	1.9365 (18)	C25—H25	0.9500
As—C11	1.9414 (19)	C26—H26	0.9500
C11—C16	1.383 (3)	C241—H24A	0.9800
C11—C12	1.392 (3)	C241—H24B	0.9800
C12—C13	1.388 (3)	C241—H24C	0.9800
C12—H12	0.9500	C31—C36	1.390 (3)
C13—C14	1.389 (3)	C31—C32	1.390 (3)
C13—H13	0.9500	C32—C33	1.387 (3)
C14—C15	1.390 (3)	C32—H32	0.9500
C14—C141	1.503 (3)	C33—C34	1.392 (3)
C15—C16	1.390 (3)	C33—H33	0.9500
C15—H15	0.9500	C34—C35	1.390 (3)
C16—H16	0.9500	C34—C341	1.501 (3)
C141—H14A	0.9800	C35—C36	1.386 (3)
C141—H14B	0.9800	C35—H35	0.9500
C141—H14C	0.9800	C36—H36	0.9500
C21—C26	1.386 (3)	C341—H34A	0.9800
C21—C22	1.389 (3)	C341—H34B	0.9800
C22—C23	1.389 (3)	C341—H34C	0.9800
C22—H22	0.9500		
			
Br^i^—Pd—Br	180.0	C22—C23—C24	120.89 (19)
Br^i^—Pd—As^i^	87.786 (7)	C22—C23—H23	119.6
Br—Pd—As^i^	92.214 (7)	C24—C23—H23	119.6
Br^i^—Pd—As	92.214 (7)	C25—C24—C23	118.14 (18)
Br—Pd—As	87.786 (7)	C25—C24—C241	120.99 (19)
As^i^—Pd—As	180.000 (6)	C23—C24—C241	120.86 (19)
C21—As—C31	101.19 (8)	C24—C25—C26	121.34 (19)
C21—As—C11	102.75 (8)	C24—C25—H25	119.3
C31—As—C11	102.44 (8)	C26—C25—H25	119.3
C21—As—Pd	116.10 (5)	C21—C26—C25	120.17 (18)
C31—As—Pd	113.54 (6)	C21—C26—H26	119.9
C11—As—Pd	118.50 (5)	C25—C26—H26	119.9
C16—C11—C12	119.31 (18)	C24—C241—H24A	109.5
C16—C11—As	121.36 (14)	C24—C241—H24B	109.5
C12—C11—As	119.32 (14)	H24A—C241—H24B	109.5
C13—C12—C11	119.97 (18)	C24—C241—H24C	109.5
C13—C12—H12	120.0	H24A—C241—H24C	109.5
C11—C12—H12	120.0	H24B—C241—H24C	109.5
C12—C13—C14	121.04 (18)	C36—C31—C32	119.32 (18)
C12—C13—H13	119.5	C36—C31—As	121.41 (14)
C14—C13—H13	119.5	C32—C31—As	119.07 (14)
C13—C14—C15	118.55 (18)	C33—C32—C31	119.87 (19)
C13—C14—C141	120.90 (19)	C33—C32—H32	120.1
C15—C14—C141	120.49 (19)	C31—C32—H32	120.1
C14—C15—C16	120.6 (2)	C32—C33—C34	121.40 (19)
C14—C15—H15	119.7	C32—C33—H33	119.3
C16—C15—H15	119.7	C34—C33—H33	119.3
C11—C16—C15	120.46 (19)	C35—C34—C33	118.05 (19)
C11—C16—H16	119.8	C35—C34—C341	121.11 (19)
C15—C16—H16	119.8	C33—C34—C341	120.79 (19)
C14—C141—H14A	109.5	C36—C35—C34	121.10 (19)
C14—C141—H14B	109.5	C36—C35—H35	119.4
H14A—C141—H14B	109.5	C34—C35—H35	119.4
C14—C141—H14C	109.5	C35—C36—C31	120.26 (18)
H14A—C141—H14C	109.5	C35—C36—H36	119.9
H14B—C141—H14C	109.5	C31—C36—H36	119.9
C26—C21—C22	119.00 (17)	C34—C341—H34A	109.5
C26—C21—As	121.09 (14)	C34—C341—H34B	109.5
C22—C21—As	119.90 (14)	H34A—C341—H34B	109.5
C23—C22—C21	120.40 (19)	C34—C341—H34C	109.5
C23—C22—H22	119.8	H34A—C341—H34C	109.5
C21—C22—H22	119.8	H34B—C341—H34C	109.5
			
Br^i^—Pd—As—C21	−142.07 (6)	Pd—As—C21—C22	52.48 (17)
Br—Pd—As—C21	37.93 (6)	C26—C21—C22—C23	−2.5 (3)
Br^i^—Pd—As—C31	101.25 (6)	As—C21—C22—C23	176.57 (15)
Br—Pd—As—C31	−78.75 (6)	C21—C22—C23—C24	2.1 (3)
Br^i^—Pd—As—C11	−19.01 (6)	C22—C23—C24—C25	0.0 (3)
Br—Pd—As—C11	160.99 (6)	C22—C23—C24—C241	−178.57 (19)
C21—As—C11—C16	16.75 (17)	C23—C24—C25—C26	−1.7 (3)
C31—As—C11—C16	121.44 (16)	C241—C24—C25—C26	176.82 (19)
Pd—As—C11—C16	−112.75 (15)	C22—C21—C26—C25	0.8 (3)
C21—As—C11—C12	−162.31 (15)	As—C21—C26—C25	−178.28 (15)
C31—As—C11—C12	−57.62 (16)	C24—C25—C26—C21	1.4 (3)
Pd—As—C11—C12	68.20 (16)	C21—As—C31—C36	89.24 (16)
C16—C11—C12—C13	−1.3 (3)	C11—As—C31—C36	−16.66 (17)
As—C11—C12—C13	177.82 (14)	Pd—As—C31—C36	−145.64 (14)
C11—C12—C13—C14	0.1 (3)	C21—As—C31—C32	−85.50 (16)
C12—C13—C14—C15	1.6 (3)	C11—As—C31—C32	168.60 (15)
C12—C13—C14—C141	−175.71 (19)	Pd—As—C31—C32	39.62 (16)
C13—C14—C15—C16	−2.2 (3)	C36—C31—C32—C33	−0.6 (3)
C141—C14—C15—C16	175.19 (19)	As—C31—C32—C33	174.29 (14)
C12—C11—C16—C15	0.7 (3)	C31—C32—C33—C34	−0.2 (3)
As—C11—C16—C15	−178.33 (15)	C32—C33—C34—C35	1.1 (3)
C14—C15—C16—C11	1.0 (3)	C32—C33—C34—C341	−176.32 (19)
C31—As—C21—C26	−5.08 (18)	C33—C34—C35—C36	−1.1 (3)
C11—As—C21—C26	100.58 (17)	C341—C34—C35—C36	176.23 (19)
Pd—As—C21—C26	−128.46 (15)	C34—C35—C36—C31	0.4 (3)
C31—As—C21—C22	175.86 (16)	C32—C31—C36—C35	0.5 (3)
C11—As—C21—C22	−78.48 (16)	As—C31—C36—C35	−174.25 (15)

Symmetry codes: (i) −*x*+1, −*y*+1, −*z*+1.

### Hydrogen-bond geometry (Å, °)

**Table:**

*D*—H···*A*	*D*—H	H···*A*	*D*···*A*	*D*—H···*A*
C32—H32···Br	0.95	2.95	3.764 (2)	144
C35—H35···Br^ii^	0.95	2.94	3.787 (2)	149

Symmetry codes: (ii) *x*−1/2, −*y*+3/2, *z*−1/2.
